# Why we still miss breast cancers: strategies for improving mammography interpretation

**DOI:** 10.1186/s13244-025-02168-2

**Published:** 2026-01-05

**Authors:** Niketa Chotai, Aishwarya Gadwal, Divya Buchireddy, Wei Tse Yang

**Affiliations:** 1 RadLink Women Imaging Center, Tan Tock Seng Hospital, Singapore, Singapore; 2 Department of Medical Imaging, University of Toronto, Toronto, ON Canada; 3 Siemens Healthineers, Mumbai, India; 4 Department of Breast Imaging, University of Texas MD Anderson Cancer Center, Houston, TX USA

**Keywords:** Missed breast cancer, Mammogram, Strategies, False negative

## Abstract

**Abstract:**

Diagnostic errors in mammography—particularly missed or delayed breast cancer detection—have a substantial impact on patient outcomes. These misdiagnoses remain a leading cause of malpractice claims in radiology, underscoring their serious clinical and legal implications. Contributing factors to errors in breast imaging include reader-related cognitive biases, lesion characteristics, patient-specific variables, and technical limitations. To address these challenges, a systematic approach is essential. Key strategies include structured error recognition, peer review processes, and robust quality assurance programs. Educational initiatives and system-level interventions—such as structured training, continuous feedback loops, and the integration of AI-driven computer-aided detection (CAD) tools—can significantly reduce diagnostic errors and enhance accuracy in breast imaging interpretation. This article aims to highlight common pitfalls in mammography, analyze root causes, and propose practical strategies for improvement. Real-life cases of missed diagnoses are included to reinforce key learning points and support radiologists in improving diagnostic precision and improving patient care.

**Critical relevance statement:**

Missed or delayed breast cancer diagnoses stem from multiple factors. A multi-pronged strategy—combining peer review, bias mitigation, education, supportive environments, and AI tools—can improve diagnostic accuracy and enhance interpretive accuracy and advance quality standards in breast imaging practice.

**Key Points:**

- Missed or delayed breast cancer diagnoses on mammography continue to be a significant source of diagnostic error with serious clinical and medico-legal consequences.
- Contributing factors to missed or delayed breast cancer diagnoses include cognitive biases, subtle lesion characteristics, patient-specific variables, and technical limitations.
- Structured peer review, double reading, and robust quality assurance programs can reduce interpretive variability and improve diagnostic performance.
- Educational initiatives and AI-driven tools, such as computer-aided detection (CAD), support error reduction and enhance accuracy in breast imaging interpretation.

**Graphical Abstract:**

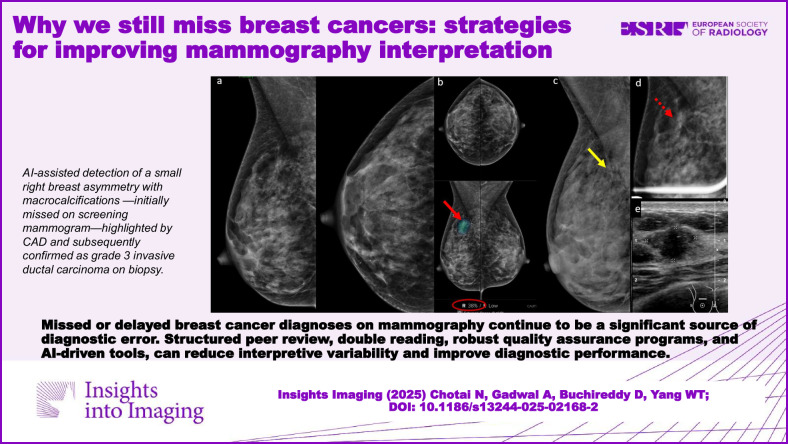

## Introduction

Breast imaging is a high-risk subspecialty within Radiology, particularly in the context of medico-legal cases involving missed cancer diagnoses. Studies show that nearly half of all missed breast cancers are retrospectively visible on prior imaging, classifying them as true perceptual errors. Whang et al reported that diagnostic errors leading to missed breast cancers are a major cause of patient morbidity, mortality and medical malpractice litigation [1]. According to Ekpo et al, 10–30% of breast cancers are misdiagnosed on screening mammography [2]. These missed diagnoses can result from a range of contributory factors, including reader-related errors, lesion-specific features, patient-related variables, and technical limitations (Table 1). This educational review explores these factors through illustrative case examples and offers practical strategies to address diagnostic blind spots and reduce error rates in mammography interpretation.

**Table 1:** Missed breast cancer: error types, causes, and mitigation strategies

	Underlying cause	Example/scenario	Suggested strategy
Reader factors			
Satisfaction of search	Premature termination after the first finding	Contralateral cancer overlooked (Fig. 1)	Structured search pattern; systematic secondary review
Anchoring bias	Fixation on initial impression	Missed additional or discordant lesion (Fig. 2)	Re-assess with a fresh perspective; seek second opinions
Availability bias	Influence of recent cases or recent literature	Overdiagnosis or missed cancer	Maintain diagnostic objectivity; focus on prevalence and clinical context
Blind spot bias	Inattention to peripheral regions	Missed axillary, inframammary, or retro-areolar lesions	Include periphery in systematic review; use supplementary views when indicated
Inattention/distraction	Fatigue, multitasking, or interruptions	Subtle cancers missed in complex or dense cases (Fig. 3)	Use structured reporting, checklists, double reading or AI-assisted workflows
Lesion characteristics			
Stable-appearing lesion	Slow-growing or indolent cancers	Low-grade DCIS or ILC with minimal interval change (Fig. 4)	Compare multiple prior studies; heightened vigilance for subtle progression
One-view abnormality	No correlate on the second view or ultrasound	Dismissed abnormality (Fig. 5)	Obtain additional views/DBT; consider biopsy/MRI if clinical suspicion persists
Isodense asymmetry	Lesion camouflaged in dense tissue	Missed cancer in dense breast (Fig. 6)	Investigate new or evolving asymmetries; add targeted ultrasound
Architectural distortion	Subtle or unfamiliar signs	Overlooked finding in dense parenchyma (Fig. 7)	Use of DBT; education on recognizing distortion patterns
Faint microcalcifications	Low conspicuity, especially in dense tissue	Missed DCIS (Fig. 8)	Optimal ambient lighting; magnification tools; digital zoom
Pseudobenign morphology	Malignant lesions mimicking benign features	Hypoechoic mass with benign rim in postmenopausal woman (Fig. 9)	Higher index of suspicion; assess contextually with age, symptoms, and risk factors
Patient variables			
Dense breast	Tissue masking of subtle lesions	Delayed diagnosis in heterogeneously dense breast (Fig. 10)	Supplement with ultrasound or MRI; consider DBT in screening
Anatomical challenges	Obesity, scoliosis, shoulder issues	Poor positioning, incomplete breast inclusion	Technologist training; use of positioning aids; post-processing enhancement
Altered breast	Post-surgical or augmented breasts	Masking or misinterpretation of changes (Fig. 11)	Obtain surgical history; interpret in context; use adjunct imaging if needed
Technical factors			
Poor technique	Inadequate positioning, exposure, or artifacts	Skin folds, poor resolution (Fig. 12)	Adhere to PGMI criteria; repeat suboptimal studies; optimize technique
Equipment limitations	Low image quality, poor contrast, or noise	Missed subtle findings	Routine QA; compliance with MQSA; prompt correction of artifacts

*DBT* digital breast tomosynthesis, *DCIS* ductal carcinoma in situ, *ILC* invasive lobular carcinoma, *PGMI* perfect good moderate inadequate (image quality criteria), *MQSA* Mammography Quality Standards Act (enacted in 1992 by the US Food and Drug Administration), *QA* quality assurance

## Reader factors

Diagnostic errors may arise from perception errors, where an abnormality is present but not recognized by the radiologist, or interpretation errors, where an identified abnormality is mischaracterized or underestimated. Radiologists’ judgment and decision-making can be influenced by several factors, including workload, time pressure, mental and emotional state, systemic challenges (such as communication breakdown and equipment issues) and cognitive biases. Among these, cognitive bias is a key factor and involves erroneous judgment, misinterpretation, and ineffective heuristics [3, 4]. Implementing structured reporting, diagnostic checklists, and awareness of cognitive bias, together with systematic feedback on outcomes and regular reflection on personal practice, are essential strategies to improve interpretive accuracy and minimize reader-related causes of missed breast cancers. Some of the most common cognitive biases leading to missed breast cancers are described below.

### Satisfaction of search

**Fig. 1:**
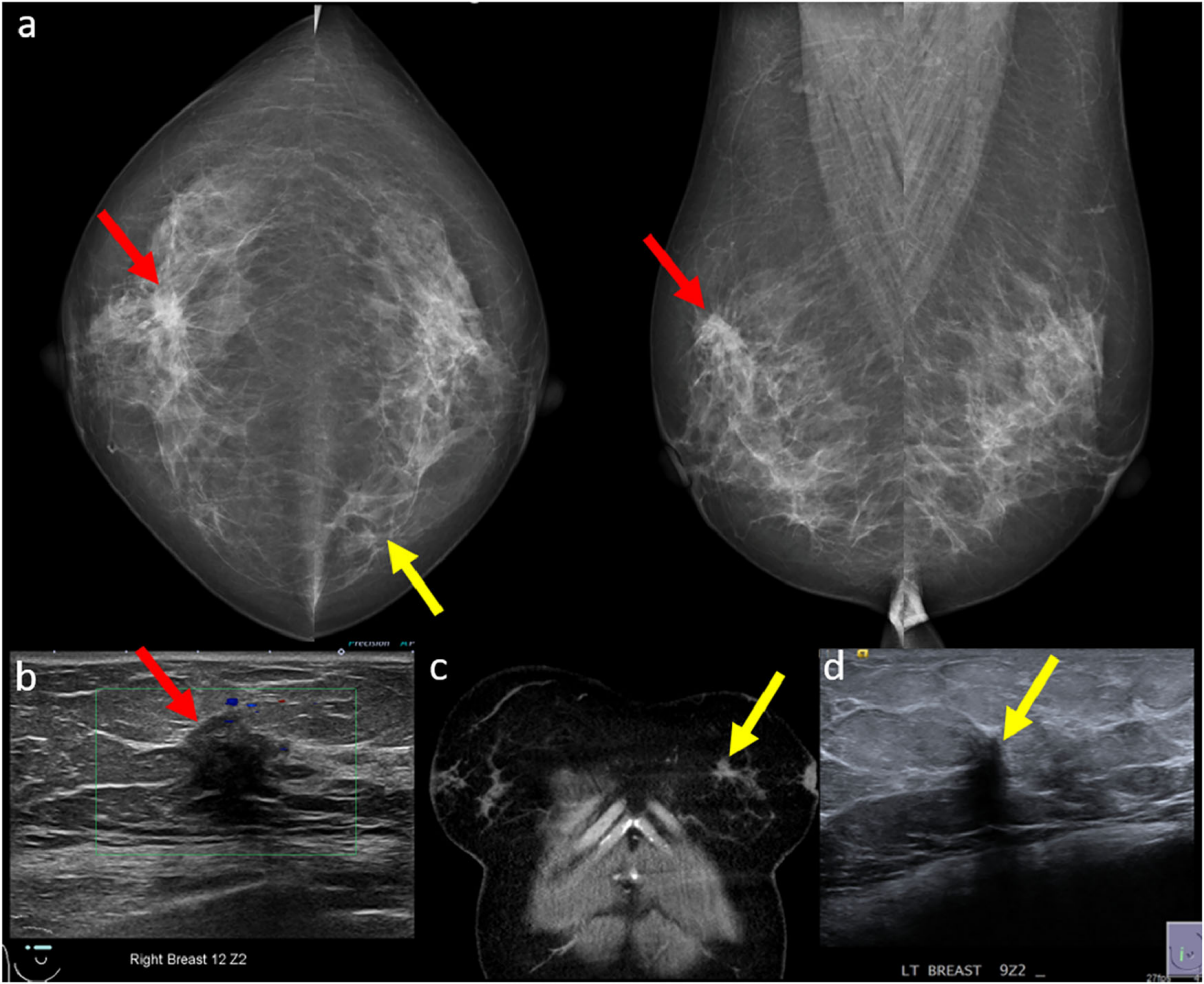
**a** Mammogram of a 48-year-old woman with a palpable right breast lump showed a spiculated high-density mass in the upper central right breast (red arrows). **b** Corresponding ultrasound showed an irregular hypoechoic mass with angular margins (red arrow) at 12 o’clock. Ultrasound-guided core biopsy revealed invasive ductal carcinoma. **c** Staging CT (coronal image) showed a spiculated mass in the contralateral (left) breast (yellow arrow). Retrospective review of the screening mammogram identified a previously overlooked asymmetry in the medial left breast (yellow arrow). **d** CT-directed breast ultrasound of the left breast demonstrated an irregular hypoechoic mass with indistinct margins and posterior shadowing at 9 o’clock (yellow arrow), corresponding to the CT finding. Ultrasound-guided core biopsy revealed invasive tubular carcinoma

One common bias in breast imaging is satisfaction of search, where the detection of an initial abnormality reduces vigilance for identifying additional findings. This is especially critical in breast imaging, as 6–60% of breast malignancies may present as multifocal or multicentric disease [5]. For example, a synchronous tumor in the contralateral breast may be overlooked after identifying a primary malignancy (Fig. 1).

**Strategies to mitigate satisfaction of search:**

- Systematic review before lesion of interest focus: Before concentrating on the primary finding, perform a structured survey of the entire study to ensure no other abnormalities are missed.
- Secondary search: Conduct an intentional second review after evaluating the primary lesion to detect additional findings and assess potential blind spots, after the primary search.
- Use of standardized checklists: Implementing structured templates can reinforce a comprehensive, consistent, and safe diagnostic approach.

### Anchoring and confirmation bias

**Fig. 2:**
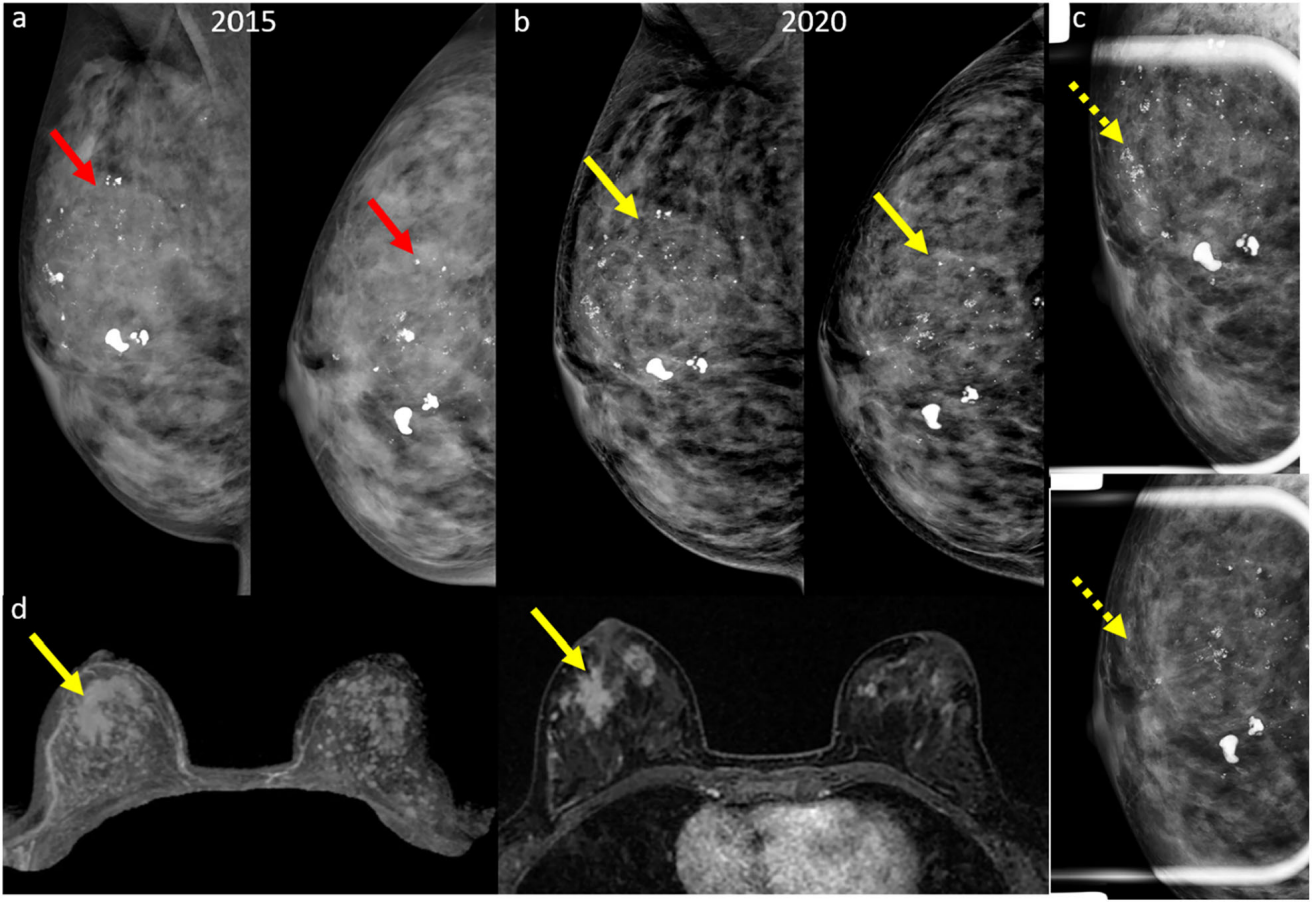
**a** Screening mammogram in a 43-year-old woman in 2015 revealed regionally distributed coarse heterogeneous microcalcifications in the right breast (red arrows), interpreted as benign. **b** In 2020, a different radiologist reviewing the screening mammogram described a slow interval increase in the number of microcalcifications and suspicious coarse heterogeneous morphology (yellow arrows). **c** Magnification views reveal fine pleomorphic microcalcifications in the upper central right breast and associated architectural distortion (dotted yellow arrows). Targeted ultrasound (not shown) did not reveal a definitive correlate, prompting a recommendation for MRI. **d** MRI demonstrated a spiculated enhancing mass (yellow arrows) in the right breast corresponding to the mammographic region of interest previously considered stable. MRI-guided biopsy revealed ductal carcinoma in situ. Patient opted for right mastectomy and final surgical histology reported 4.5 cm intermediate-grade ductal carcinoma in situ associated with lobular carcinoma in situ

Anchoring bias occurs when a radiologist remains fixed on an initial diagnosis, even when new and conflicting information becomes available [6]. This often results from overreliance on previous reports—whether by the same or another radiologist—which can influence the interpretation of current findings (Fig. 2). Mislocalization errors, such as assuming a lesion seen only on the MLO view is lateral rather than medial, represent another cause of missed cancers.

Confirmation bias arises when a radiologist unconsciously seeks evidence that supports a pre-existing hypothesis, rather than objectively reassessing the case [7]. This bias stems from a natural tendency to seek affirmation over precision [8].

**Strategies to mitigate this error:**

- Approach each case with a fresh, independent perspective before reviewing previous reports, reducing the risk of anchoring bias.
- Encourage diagnostic flexibility by actively considering alternative differential diagnoses and seeking a second opinion when appropriate.

### Availability bias

Availability bias occurs when a radiologist’s diagnostic reasoning is disproportionately influenced by recently encountered cases-whether from personal experience, medical literature, or professional meetings. This bias can lead to an overestimation of certain conditions while neglecting less common but plausible diagnoses. For instance, a radiologist who recently diagnosed multiple cases of mastitis may be predisposed to interpret similar findings as infection, potentially overlooking a malignancy. Such cognitive replay loops can result in both overdiagnosis and missed diagnoses, ultimately compromising patient care [4].

**Strategies to mitigate this bias:**

- Maintain awareness of the actual frequency of uncommon diagnoses to avoid overweighing more common ones.
- As suggested by Itri et al, regular evaluation of peer-reviewed literature and conferring with colleagues can help counteract availability bias [4].

### Blind spot bias

Blind spot bias occurs when radiologists overlook subtle lesions located in areas that are not routinely scrutinized-such as the inframammary folds, axillary regions, or retro-areolar areas. These regions are often outside the standard search pattern, increasing the chance of missed findings. As Drew et al highlighted, this bias can stem from insufficient training in evaluating these locations. Contributing factors include patient body habitus, suboptimal positioning, tissue overlap, and limited visibility in standard mammographic views [8, 9].

**Strategies to reduce blind spot bias:**

- Use a systematic search pattern that consistently includes commonly overlooked areas.
- Obtain supplementary projections when standard projections do not fully visualize suspicious regions.
- Stay mindful of personal perceptual tendencies and habitual blind spots.

**Common blind spots on mammography include:**

- Retroglandular clear space on CC view (“No-man’s land”)
- Area adjacent and anterior to the pectoralis major muscle on MLO view (“Milky Way”)
- Inner half of the breast on CC view
- Retro-areolar region [8, 9].

### Inattention bias

Inattention bias arises when radiologists unintentionally miss abnormalities due to distractions or reduced focus. This can result from fatigue, time pressure, multitasking, or digital interruptions such as mobile messaging apps and social media [10]. These distractions are especially challenging when interpreting screening mammograms, where detecting subtle signs of breast cancer requires sustained attention. Additionally, complex cases with multiple findings may draw focus away from smaller, less conspicuous yet clinically significant abnormalities.

**Strategies to reduce inattention bias:**

**Fig. 3:**
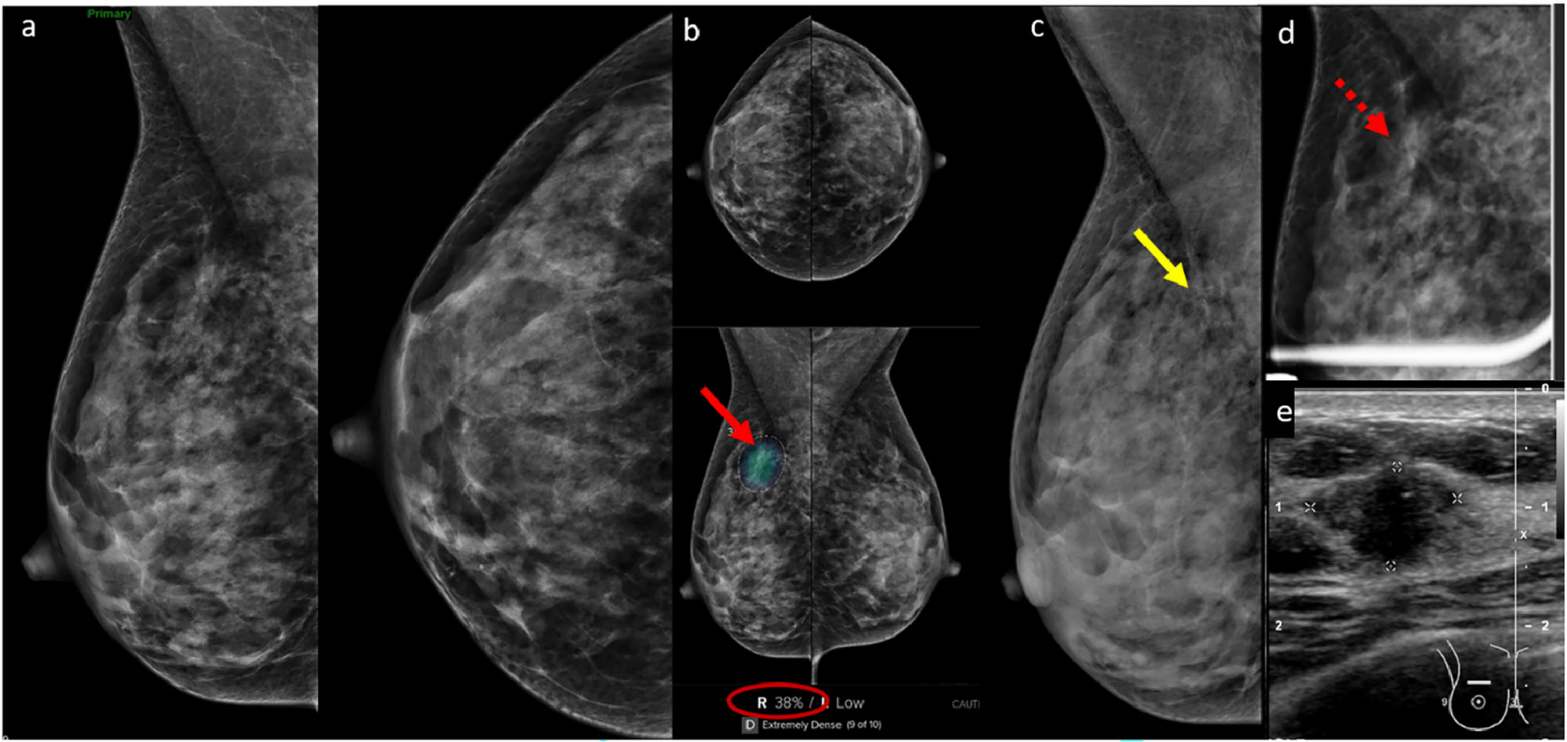
**a** Screening mammogram in a 44-year-old woman initially interpreted as normal by the radiologist on a particularly busy day. **b** Subsequent CAD analysis using Lunit INSIGHT MMG v1.1.7.1 annotated an isodense asymmetry with associated microcalcifications (red arrow) in the upper posterior right breast, seen on MLO projection only. CAD flagged this area (red arrow) with a 38% likelihood of malignancy (red circle). **c** Retrospective review of a prior mammogram from a year ago revealed a small group of round microcalcifications in linear distribution (yellow arrow) in the area of CAD interest, not previously described. **d** Magnification imaging showed persistent asymmetry (dotted red arrow) with subtle architectural distortion and microcalcifications in the area of concern. **e** Ultrasound shows a corresponding irregular, non-parallel, hypoechoic mass with indistinct margins. Ultrasound-guided biopsy revealed grade 3 invasive ductal carcinoma

- Apply a consistent search strategy and use checklists or structured reporting templates.
- Engage in regular training and implement audit reviews to refine focus [11].
- Minimize distractions by limiting device use and social media during reading sessions.
- Take short mental breaks to avoid cognitive fatigue.
- Use double-reading strategies or integrate AI as a second reader. AI maintains consistent performance that is unaffected by fatigue or distraction and can help to flag subtle findings (Fig. 3).

## Lesion characteristics

Certain intrinsic features of breast lesions can make them particularly difficult to detect or interpret on mammograms. Factors influencing visibility include lesion density, which may be harder to judge with modern post-processing, and margins, where spiculation suggests slower-growing cancers, while higher-grade tumors often appear deceptively smooth or ill-defined, especially when not surrounded by fat. Adequate fat contrast is therefore important, as limited fat reduces lesion conspicuity even on DBT. Importantly, high-grade, rapidly growing cancers may mimic benign masses with circumscribed margins, making them prone to misinterpretation. As highlighted by Blanks et al, small high-grade tumors are more likely to be missed on screening mammograms compared to low-grade tumors of similar size [12].

Below, we examine key lesion characteristics that contribute to diagnostic challenges:

### Apparently stable-appearing lesions

**Fig. 4:**
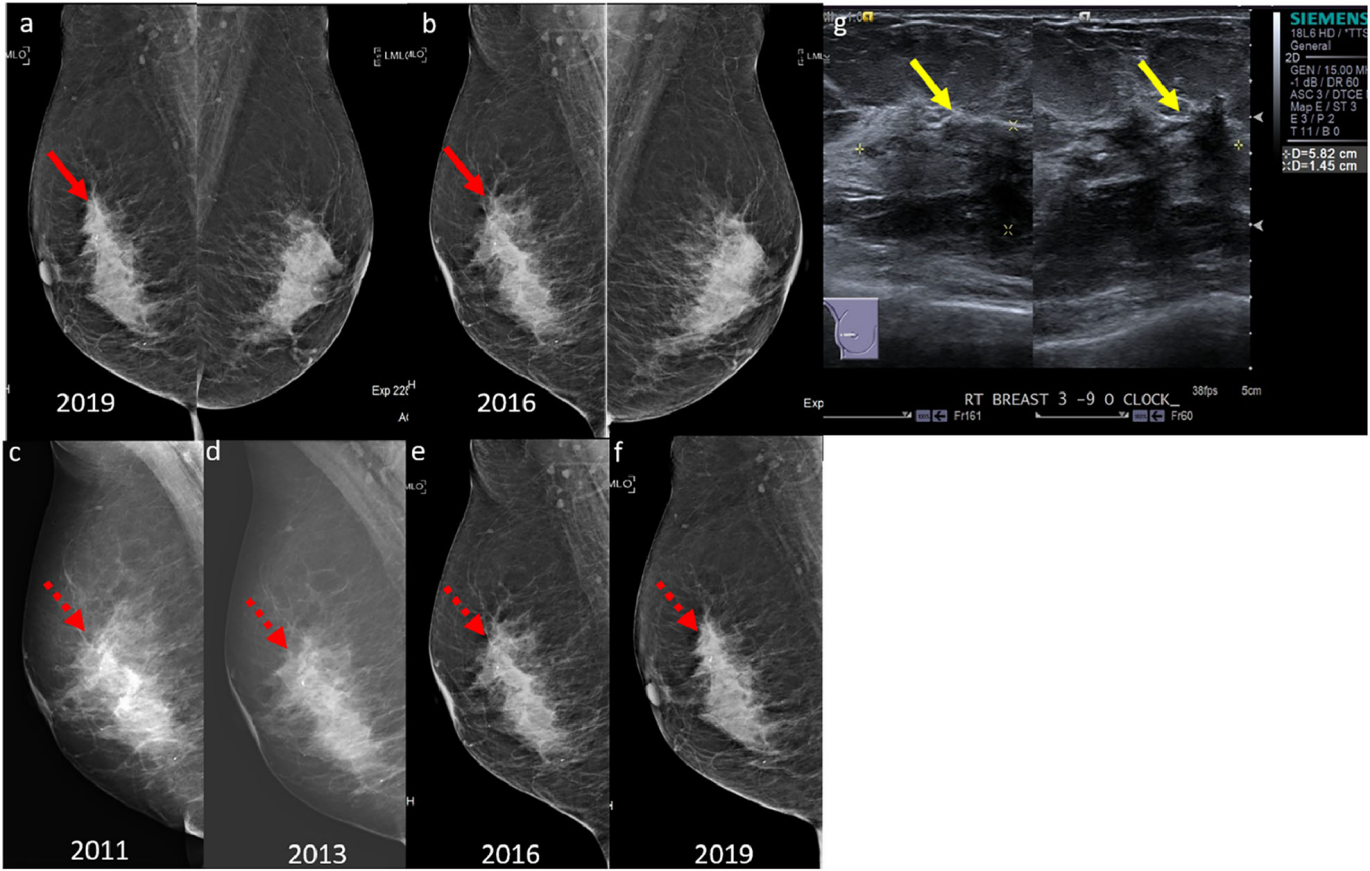
**a** A 49-year-old woman presented for an annual screening mammogram in 2019. Bilateral MLO mammograms reveal dense fibroglandular tissue with possible architectural distortion in the upper right breast (red arrow). **b** Comparison with prior mammograms dating back to 2016 showed stability of this finding. **c**–**f** Further comparison with multiple prior mammograms dating back to 2011 revealed progressive, but subtle increased conspicuity (dotted red arrows) of the architectural distortion. **g** The patient was recalled for a targeted ultrasound that revealed an irregular, hypoechoic non-mass lesion (yellow arrows) with mixed posterior features. Ultrasound-guided biopsy revealed grade 1 invasive lobular carcinoma. The case highlights the insidious and slow-growing nature of invasive lobular carcinoma, which can mimic stability over short intervals and be challenging to detect on mammography, especially in dense breasts

Lesions that appear stable across serial mammograms are often interpreted as benign, with an estimated malignancy risk of less than 2%. On average, malignant tumors double in size on mammography over approximately 212 days [13]. However, radiographic stability alone does not always confer benignity. Certain slow-growing cancers—such as low-grade ductal carcinoma in situ or invasive lobular carcinoma—can remain radiographically unchanged for extended periods, creating a false sense of reassurance. These malignancies can often grow in a diffuse or infiltrative pattern rather than forming distinct masses, making them particularly challenging to detect based on stability alone (Fig. 4).

**Strategies to reduce diagnostic error:**

- Prioritize suspicious morphological features over apparent imaging stability in guiding decisions regarding further evaluation.
- Compare multiple prior mammograms—not just the most recent study—to identify subtle or gradual changes that may signal malignancy.

### Lesions with one-view abnormality

**Fig. 5:**
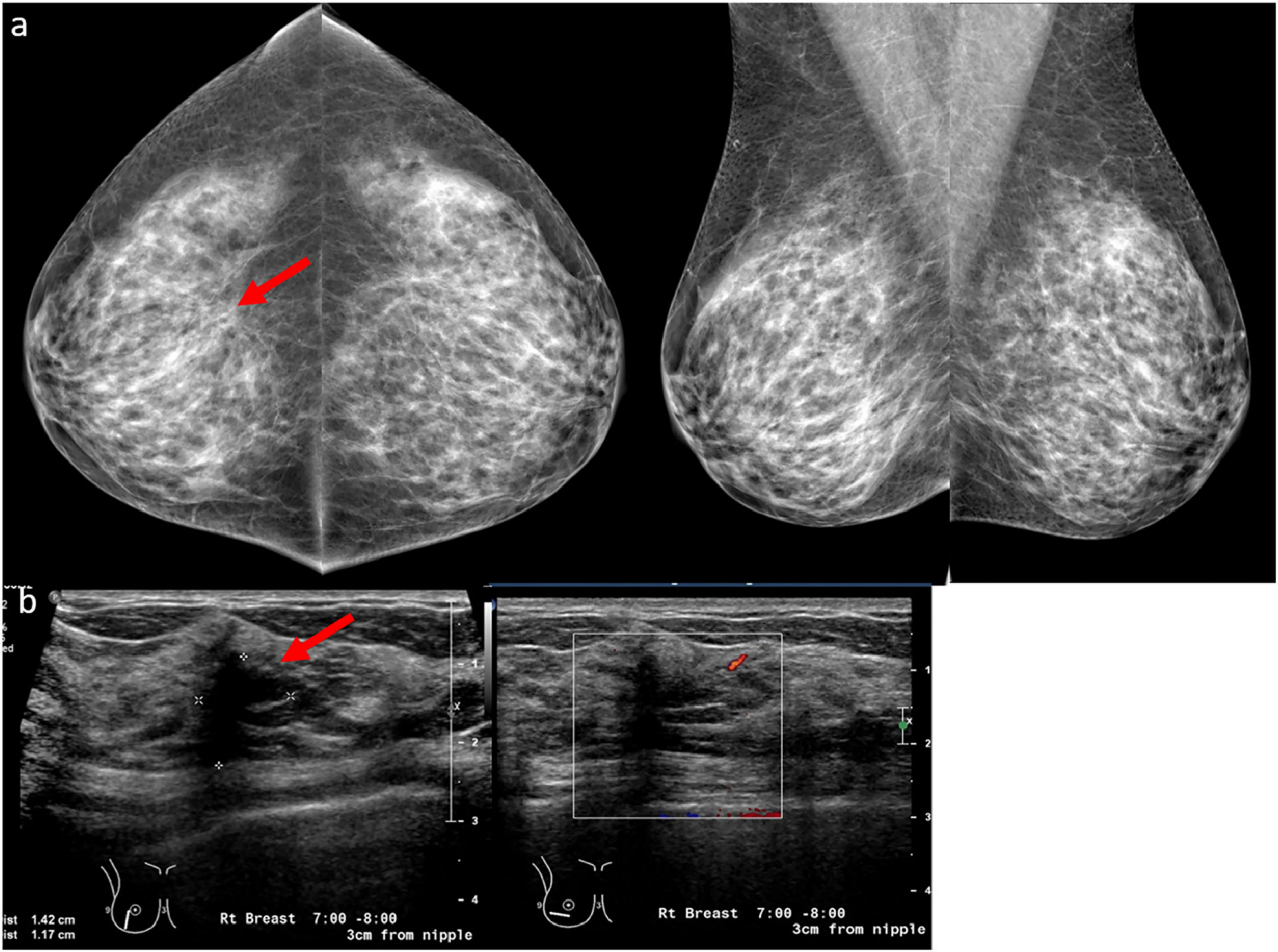
**a** CC and MLO screening mammograms in a 41-year-old woman revealed a subtle architectural distortion in the central right breast seen on the CC view only (red arrow). This finding was not reported by one reader. However, the second reader identified the abnormality and recommended a recall for further assessment. At our institution, all screening mammograms are routinely interpreted by two independent readers. **b** Targeted ultrasound revealed a focal hypoechoic non-mass lesion (red arrow) in the corresponding right breast, with associated posterior shadowing and internal vascularity. Ultrasound-guided biopsy revealed grade 1 invasive ductal carcinoma

Lesions that appear on only one of the two standard mammographic views are often misinterpreted as normal tissue or dismissed as benign asymmetry [1]. While such findings may frequently represent benign lesions, studies indicate that 9–38% of breast cancers can present in a single view only, leading to a significant risk of missed diagnoses [14]. This can occur due to factors such as dense breast tissue, suboptimal imaging, poorly defined masses, or desmoplastic reactions that obscure lesion margins (Fig. 5).

**Strategies to reduce diagnostic oversight:**

- Utilize additional imaging—such as spot compression, extended views, tomosynthesis, or ultrasound—when a finding is visible on one view only.
- Pay special attention to asymmetries that correlate with palpable abnormalities.
- Do not rely solely on a negative ultrasound. If uncertainty remains, proceed with biopsy or functional imaging like contrast-enhanced mammogram (CEM) or MRI for further evaluation.

### Isodense asymmetry

Most isodense asymmetries are benign and often related to normal variations in tissue distribution. However, any new, enlarging, or persistent asymmetry—especially when accompanied by architectural distortion or microcalcifications—should raise suspicion for malignancy. These asymmetries can be particularly challenging to detect because they blend with surrounding fibroglandular tissue, making them less conspicuous than high-contrast abnormalities such as calcifications or well-defined masses.

**Fig. 6:**
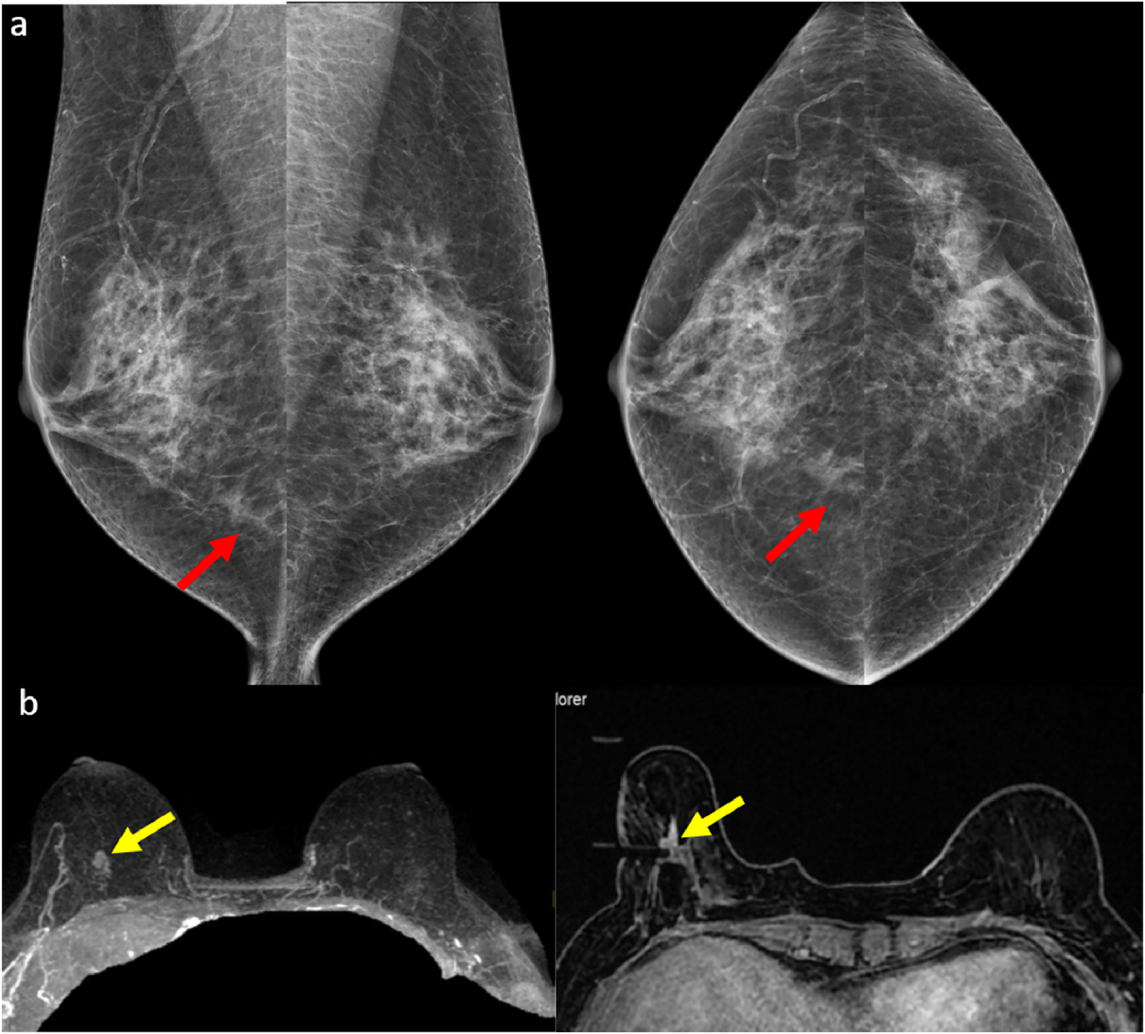
**a** MLO and CC screening mammograms in a 52-year-old woman demonstrated an isodense focal asymmetry in the lower inner right breast, projected over the retromammary fat (red arrows). This finding was not reported by the first reader, likely due to its location at the periphery of the image, and was reported by the second reader. Assessment with ultrasound (not shown) was unremarkable. **b** Breast MRI was subsequently performed, and the axial maximum intensity projection image revealed an irregular enhancing mass in the lower right breast, corresponding to the mammographic asymmetry (yellow arrows). Axial image obtained during MRI-guided biopsy, which revealed histopathology of grade 2 invasive lobular carcinoma

Detection is especially difficult in dense breast tissue, where normal parenchyma may obscure subtle changes (Fig. 6). Without meticulous comparison with prior mammograms, small changes in density or developing asymmetries can easily be missed [15]. Even minor increases in density over time may indicate early-stage breast cancer. The positive predictive value of a developing asymmetry is approximately 27%, most frequently correlating with invasive carcinoma or ductal carcinoma in situ, thus warranting histological confirmation when suspicion arises [16, 17].

**Strategies to reduce diagnostic errors:**

- Use a consistent, systematic search pattern to assess across all areas of the breast.
- Employ magnification, spot compression views, digital breast tomosynthesis (DBT), and ultrasound to differentiate true asymmetries from tissue overlap.
- Remember that a new or developing asymmetry warrants further investigation—even in the absence of an ultrasound correlate. Biopsy or MRI/CEM should be considered when uncertain.

### Subtle architectural distortion

**Fig. 7:**
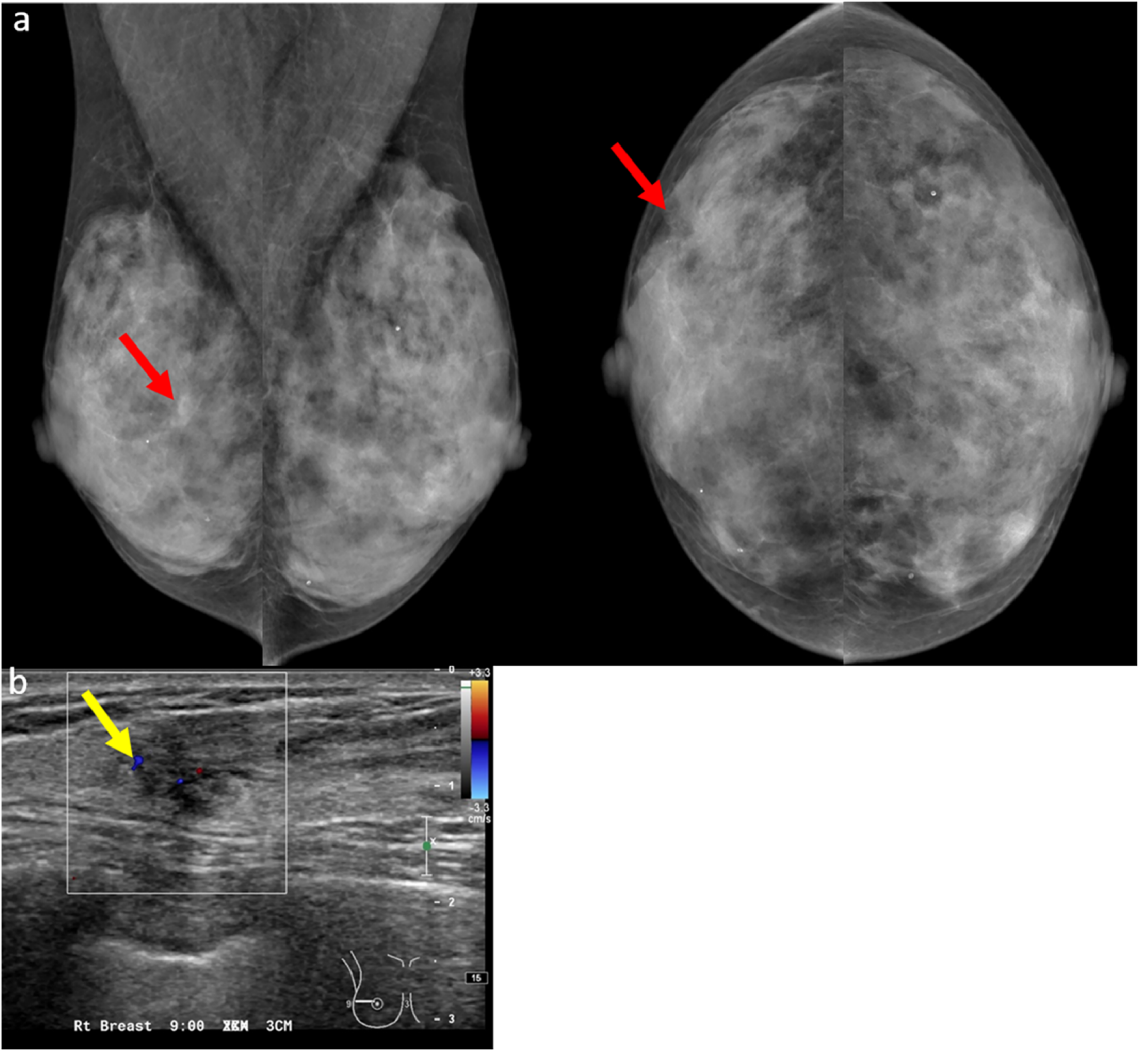
**a** First screening mammogram in a 43-year-old woman revealed a subtle architectural distortion in the outer central aspect of the right breast (red arrows) in otherwise extremely dense breast tissue. This finding was missed by one of the readers but detected by the second reader, who recalled the patient for further evaluation. Notably, there was a gentle retraction of tissue at the anterior mammary fat on the CC view, while the abnormality was difficult to appreciate on the MLO view due to overlapping dense breast tissue. **b** Targeted ultrasound revealed a correlating irregular hypoechoic mass in the same region (yellow arrow). Core biopsy confirmed the diagnosis of grade I invasive ductal carcinoma

Subtle architectural distortion is one of the more challenging findings to detect on 2D mammography, particularly in women with dense breast tissue. Detection becomes more difficult when the distortion is present only on a single view or lacks associated features such as a mass or calcifications. These findings can easily be overlooked, especially by less experienced radiologists (Fig. 7). Architectural distortion can result from benign conditions such as radial scars and complex sclerosing lesions, or from malignancies like invasive carcinoma and ductal carcinoma in situ. Approximately 12–45% of missed breast cancers present as architectural distortion [18].

**Strategies to reduce diagnostic errors:**

- Digital breast tomosynthesis (DBT) has markedly improved the detection of architectural distortion [19].
- Carefully evaluate natural junctions such as anterior and posterior mammary fat lines. Pay close attention to tethering or disruption of the normal breast architecture, particularly in dense breasts. Comparison with prior studies may help reveal early changes.
- Engage in peer learning—participation in peer review and reviewing teaching files, or double-reading sessions can enhance pattern recognition.
- Use ultrasound or MRI to search for correlates. When suspicion persists, perform a biopsy-preferably under DBT guidance-for definitive histological diagnosis.

### Faint grouped microcalcifications

**Fig. 8:**
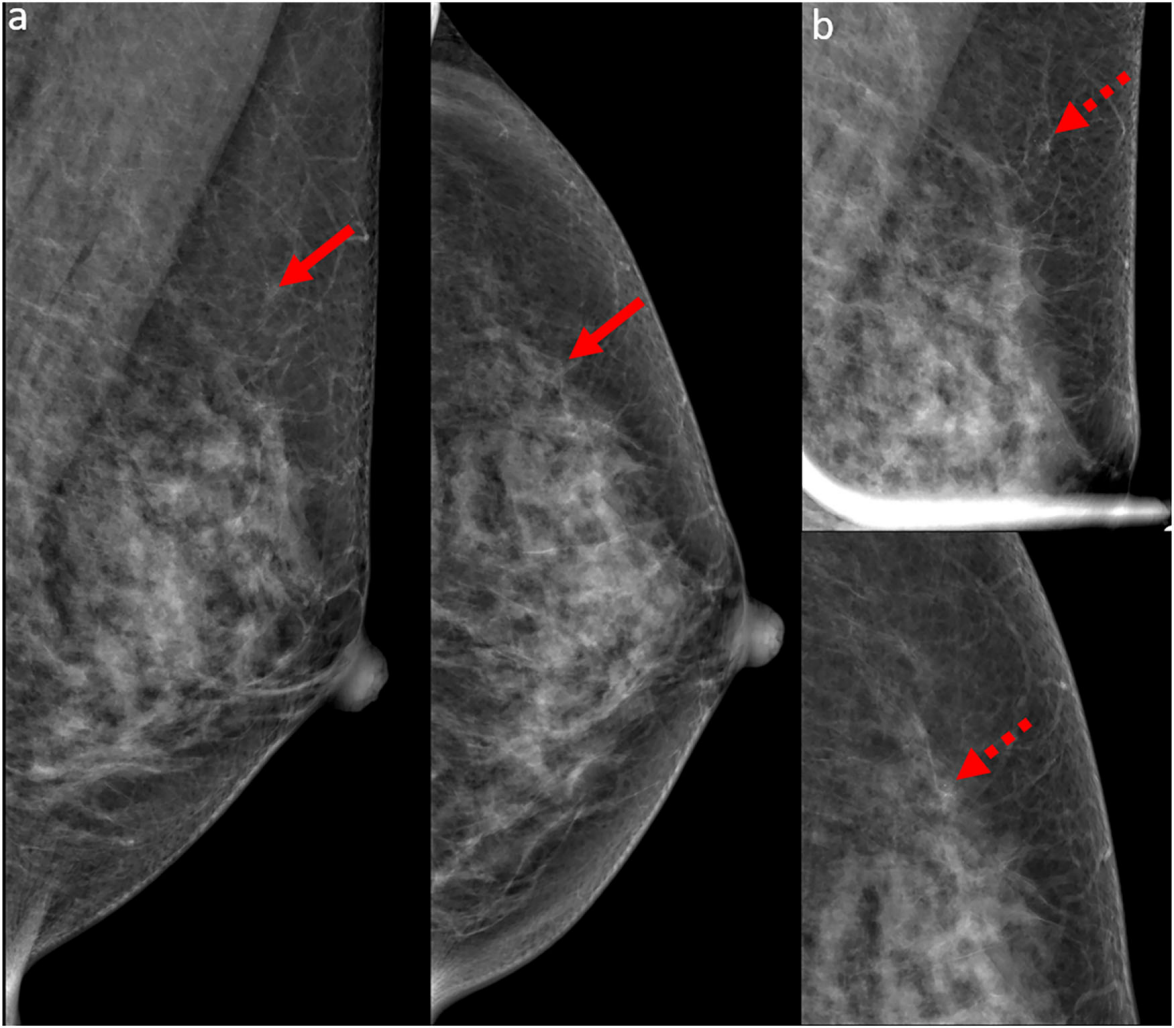
**a** CC and MLO screening mammograms in a 42-year-old woman reveal a small group of amorphous microcalcifications (red arrows) in the upper outer left breast. This finding was missed by one reader and recalled by the second reader. **b** Magnification imaging (dotted red arrows) demonstrates grouped fine pleomorphic microcalcifications. Stereotactic biopsy revealed intermediate-grade ductal carcinoma in situ

Faint groups of microcalcifications can be challenging to detect on screening mammograms due to their subtle appearance—especially when they are faint or located within dense breast tissue or at the edges of the image. The challenge increases when these calcifications are visible on only one view or are obscured by overlapping structures (Fig. 8).

**Strategies to reduce detection errors:**

- Ensure optimal viewing conditions, utilizing high-resolution monitors with appropriate luminance and contrast, and dimmed ambient lighting [20].
- Use digital magnification views and adjust window/level settings, particularly in dense breasts.
- Consider double reading or incorporating AI-CAD tools to help flag subtle or overlooked calcifications [21].

### Lesions with pseudobenign morphology

**Fig. 9:**
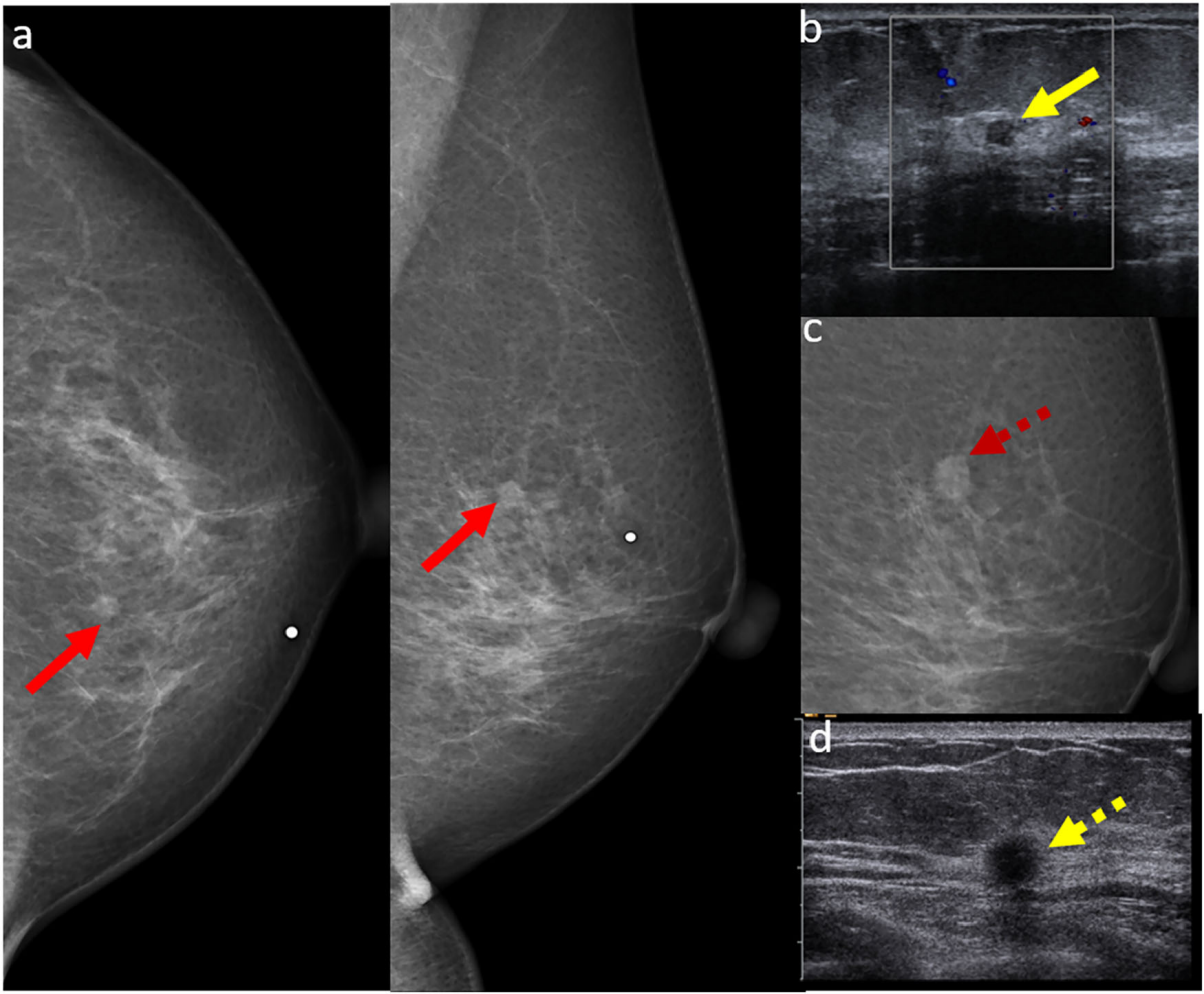
**a** A 56-year-old woman underwent baseline screening mammography. CC and MLO left mammograms demonstrated a circumscribed isodense mass with indistinct margins in the left breast (red arrows). **b** Workup ultrasound described an anechoic circumscribed subcentimeter mass (yellow arrow) corresponding to the mammographic finding interpreted as a cyst. In light of the patient’s age, it was categorized as BI-RADS 3, and a short-term follow-up was recommended. **c** Six-month follow-up MLO mammogram (CC view not shown) showed an interval increase in size of the mass with irregular margins (red dotted arrow). **d** Six-month follow-up ultrasound demonstrated an irregular hypoechoic mass with microlobulated margins and posterior shadowing (dotted yellow arrow), BI-RADS 4, suspicious for malignancy. Ultrasound-guided core biopsy revealed grade 3 triple-negative breast cancer

Some breast cancers mimic benign features, increasing the risk of misclassification and delayed diagnosis. These malignancies may appear on mammography with circumscribed margins, an oval or round shape, and an isodense mass that is indistinguishable from surrounding tissue. On ultrasound, they may exhibit similar benign features—such as circumscribed margins and posterior acoustic enhancement—creating the impression of a complicated cyst, as illustrated in Fig. 9 [22].

**Strategies to reduce diagnostic errors:**

- Be especially cautious when evaluating circumscribed masses in postmenopausal women, where new lesions are more likely to be malignant. Biopsy should be considered for any new or changing mass, even if the morphology appears benign.
- Examine additional features such as margins, the presence of an echogenic rind, and associated architectural distortion that might indicate a pseudobenign lesion.

## Patient variables

Patient characteristics—including age, breast density, anatomical variants, prior surgeries, and physiological changes such as menopausal status or hormone replacement therapy use—can significantly influence lesion visibility and the likelihood of diagnostic error. As breast tissue appearance on mammography evolves over time, incorporating relevant background information is essential to aid interpretation and improve cancer detection.

### Dense breasts

**Fig. 10:**
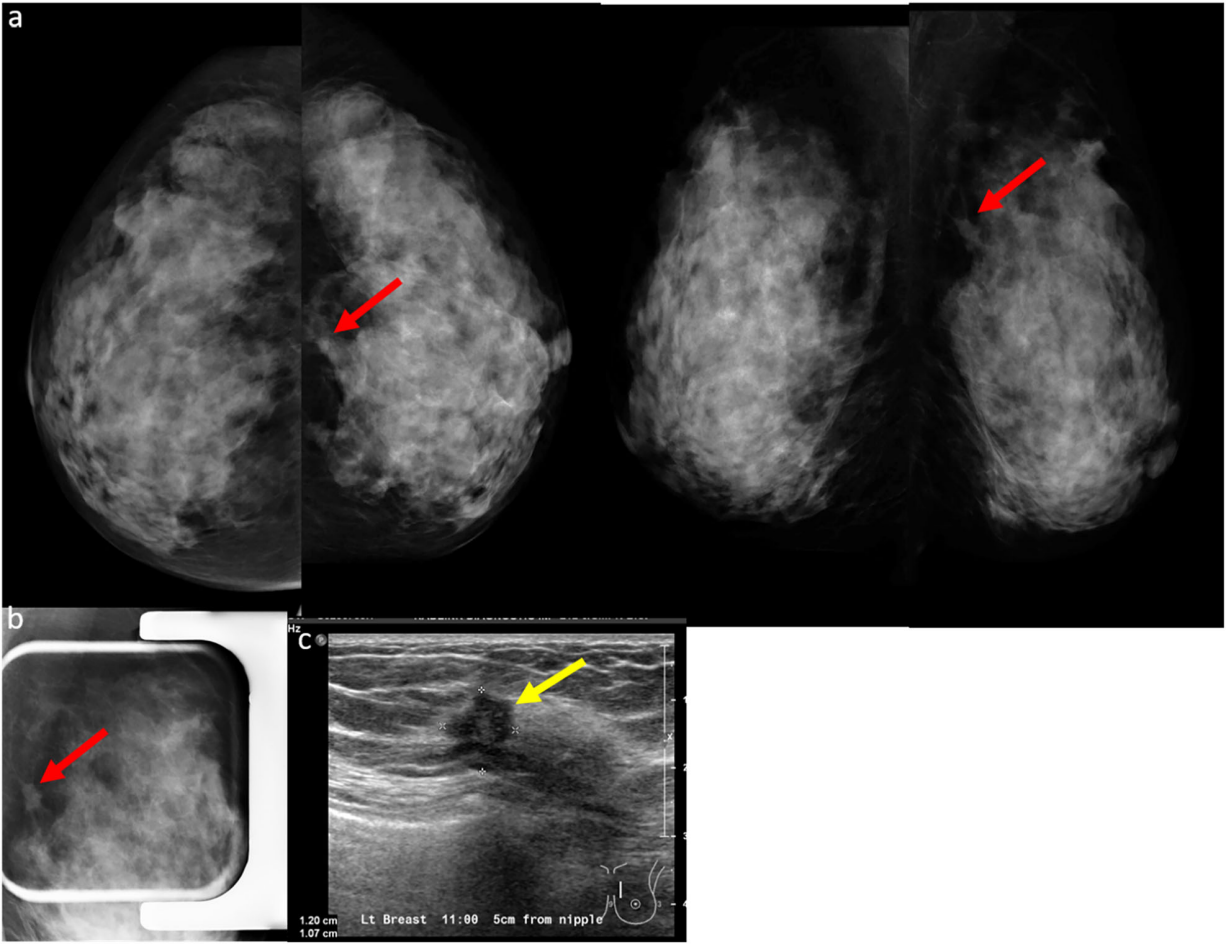
**a** A 45-year-old woman underwent a baseline screening mammogram, which was interpreted as normal by one reader. A second reader identified a possible isodense mass in the upper inner quadrant of the left breast, projected over the retromammary fat (red arrows, left CC and MLO mammograms). **b** Spot compression left lateral-medial mammogram demonstrated an irregular mass with spiculated margins (red arrow) in the area of concern. **c** Targeted ultrasound demonstrated a correlating irregular hypoechoic mass with indistinct margins (yellow arrow). Ultrasound-guided core biopsy revealed invasive ductal carcinoma

Breast density not only elevates the risk of developing breast cancer but also increases the likelihood of the cancer being missed on mammography. Breast density is one of the most important patient-related factors impacting mammographic sensitivity. There is a well-established inverse relationship between breast density and the ability of mammograms to detect breast cancer. Dense fibroglandular tissue can mask or obscure malignancies due to overlapping structures, leading to false-negative results and potentially delayed diagnosis and treatment [23]. Studies in Asian populations have reported that approximately 70–80% of women have dense breast tissue [24] (Fig. 10).

**Strategies to reduce errors:**

- Digital breast tomosynthesis (DBT) improves cancer detection, particularly in dense breasts, with an additional 2.3 to 2.7 cancers detected per 1000 screens [25].
- Supplementary breast ultrasound screening can increase cancer detection by 1.8 to 4.6 additional cancers per 1000 women with dense breasts screened [26].
- The DENSE trial, a large-scale randomized controlled study, demonstrated that adding biennial MRI screening to mammography in women with extremely dense breasts significantly reduced interval cancer rates by over 80%. Similarly, the BRAID trial compared supplemental imaging modalities—abbreviated MRI, automated breast ultrasound (ABUS), and contrast-enhanced mammography (CEM)—to standard mammography in women with dense breasts. Interim results indicated that both abbreviated MRI and CEM detected more cancers than ABUS, with abbreviated MRI identifying 17.4 cancers per 1000 exams and CEM detecting 19.2 cancers per 1000 exams [27–29].

### Anatomical and postural challenges

Certain physical conditions—such as pectus excavatum (pigeon chest), scoliosis, or frozen shoulder—can interfere with proper positioning, angulation, or breast compression during mammography. These limitations can result in suboptimal images with incomplete visualization of breast tissue, increasing the risk of missed abnormalities. Obesity introduces additional imaging challenges: abdominal contours may obscure the inframammary fold, and greater tissue thickness can attenuate X-rays and reduce image quality. Longer exposure times may also increase the likelihood of motion artefacts [30].

**Strategies to address these challenges:**

- Adapt imaging with alternative positioning techniques, larger paddles, extra views, or supplementary modalities like ultrasound and MRI.
- Ensure technologists are trained to adjust for challenging anatomy and use appropriate compression and exposure techniques (e.g., adjusting kilovoltage).
- Enhance patient comfort with props or cushions and communicate clearly to reduce anxiety and motion during imaging.
- Apply post-processing tools, including motion correction, to improve image clarity.

### Postoperative or augmented breast

**Fig. 11:**
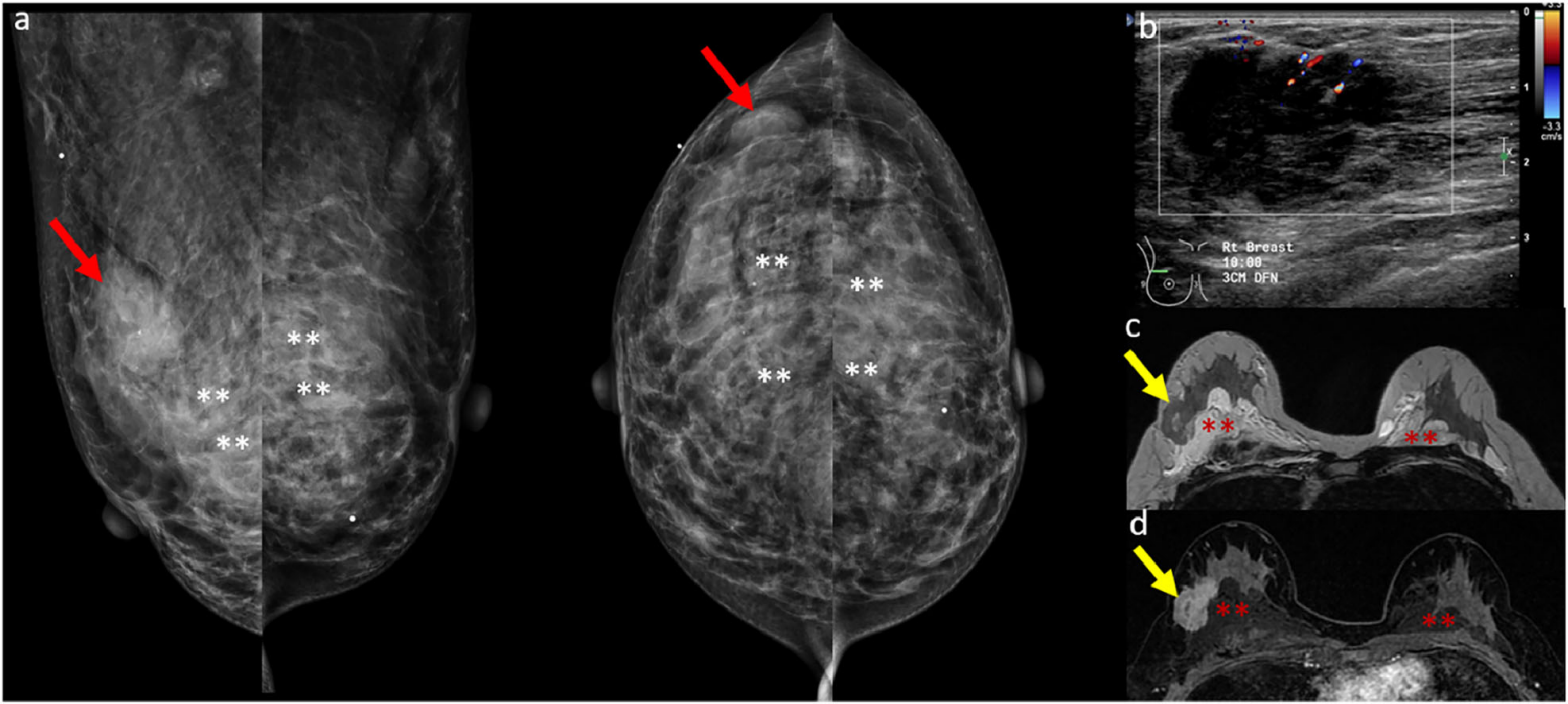
**a** MLO and CC mammograms in a 40-year-old woman revealed a focal asymmetry in the upper outer quadrant of the right breast (red arrows) adjacent to isodense polyacrylamide gel (white asterisks) previously injected for breast augmentation. **b** Ultrasound showed an irregular hypoechoic mass with indistinct margins and internal vascularity in the upper outer quadrant of the right breast that correlated with the mammographic abnormality. Imaging features were deemed suspicious, and a biopsy was recommended. **c** Axial T2-weighted non-fat-saturated and (**d**) post-contrast T1-weighted fat-saturated MRI images revealed an irregular enhancing mass (yellow arrows) in the upper outer quadrant of the right breast. Ultrasound-guided biopsy revealed grade 3 invasive ductal carcinoma. Bilateral non-enhancing, T2 hyperintense fluid collections in the retroglandular spaces (red asterisks) correspond to residual polyacrylamide gel from previous augmentation

Mammographic interpretation is more complex in patients with prior surgery or breast augmentation. Post-surgical changes—such as scarring, fat necrosis, calcifications, and architectural distortion—can mimic malignancy. Implants, particularly in the subglandular position, can obscure portions of the breast, limiting visualization of posterior or deep parenchyma. Capsular contracture may further distort normal anatomy. In patients with free filler injections, the foreign material can obscure potential lesions, increasing the risk of missed diagnoses (Fig. 11) [31].

**Strategies to improve interpretation:**

- Obtain detailed clinical history, including surgical timelines and histopathology reports for margin assessment.
- Compare current images with prior studies, especially preoperative mammograms when available.
- Perform Eklund (implant displacement) views to better visualize native breast tissue in patients with breast implants.
- Supplementary imaging with ultrasound and MRI may be helpful in patients with free silicone filler injections.

## Technical limitations

Imaging quality and technical execution can significantly impact the radiologist’s ability to detect and accurately characterize lesions. Improper positioning, inadequate compression, motion artefacts, and suboptimal exposure parameters can obscure subtle findings or distort normal anatomy. Additionally, nonadherence to standard imaging protocols may lead to incomplete evaluation—especially in patients with implants or postoperative changes [32].

### Poor technique

High-quality image acquisition is essential in mammography, as early-stage breast cancers often present with subtle radiographic findings. Image quality depends on several technical factors, all of which must be optimized to ensure accurate diagnosis and early detection.

**Fig. 12:**
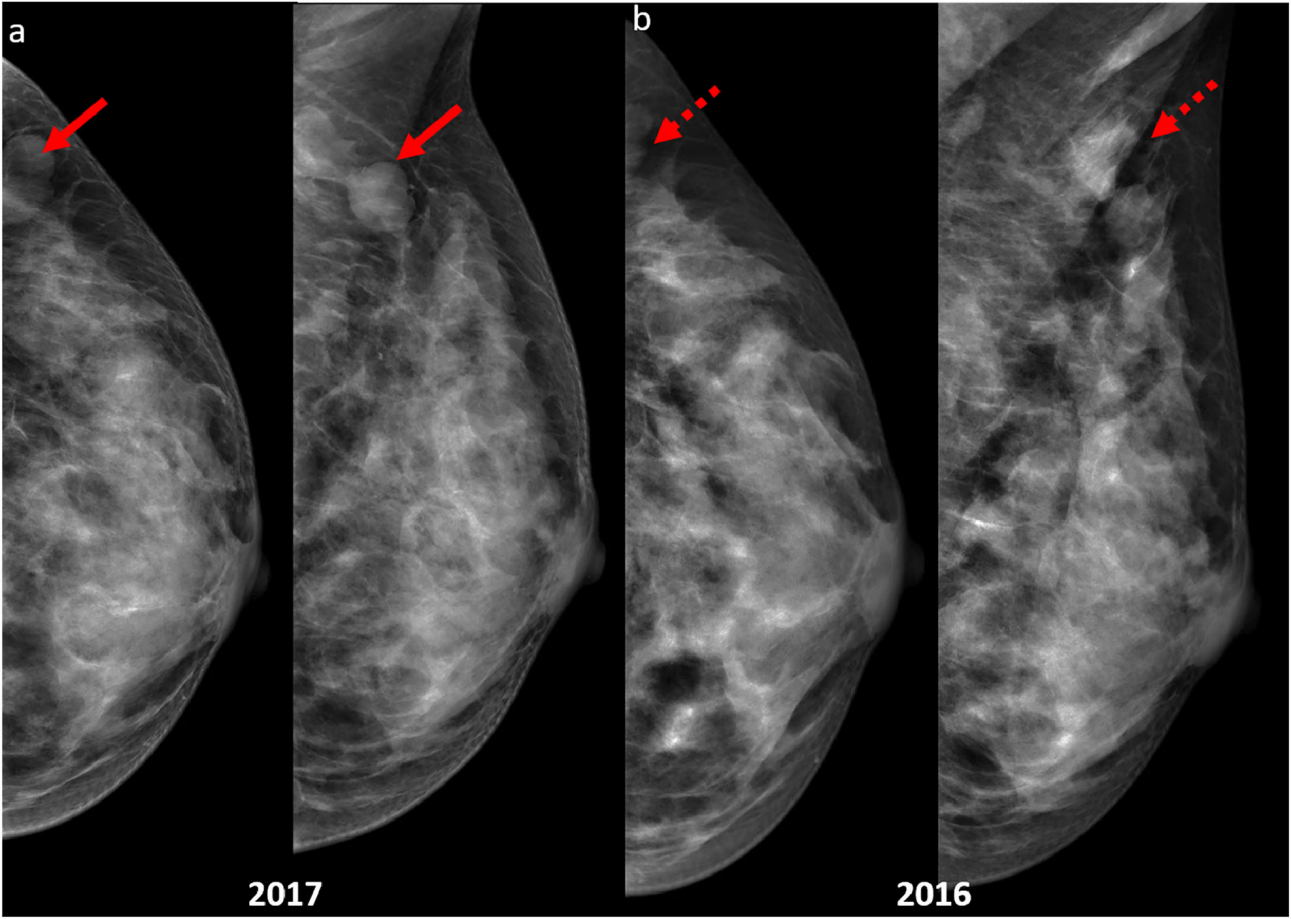
**a** CC and MLO screening mammograms in a 51-year-old woman revealed a bilobed mass with obscured margins in the left axillary tail region (red arrows) in 2017. Further assessment and biopsy confirmed the diagnosis of invasive papillary carcinoma. **b** Retrospective review of the 2016 CC and MLO screening mammograms (1 year prior) showed incomplete imaging of this mass that was partially excluded on the craniocaudal (CC) view and obscured by a skin fold on the mediolateral oblique (MLO) view (dotted arrows), leading to a negative mammogram report and missed diagnosis

Proper positioning is particularly important to fully image the breast, particularly the posterior regions, where malignancies may be hidden. Avoidance of artefacts such as skin folds and motion blur also minimizes the risk of missed lesions (Fig. 12).

Compression improves tissue separation, enhances contrast, and increases image sharpness, for the visualization of suspicious small subtle masses and microcalcifications. However, the degree of compression must be carefully balanced; excessive compression may paradoxically reduce cancer detection rates, as shown by Hudson et al [33].

Exposure settings are equally critical. Overexposure may obscure low-contrast lesions, while underexposure can compromise visibility of fine details—both potentially leading to missed cancers [34].

**Strategies to improve technique:**

- Follow PGMI (Perfect, Good, Moderate, Inadequate) criteria to ensure optimal positioning and comprehensive visualization of fibroglandular tissue. Clear depiction of the retromammary fat on both craniocaudal (CC) and mediolateral oblique (MLO) projections indicates sufficient posterior coverage.
- Utilize automatic exposure control to adjust for varying breast densities.
- Ensure technologists are trained in creative positioning techniques such as off-angle or step oblique views [22].
- Do not accept suboptimal images—repeat inadequate views when necessary.

### Poor equipment

The quality of the mammography unit itself plays a critical role in diagnostic efficacy. Outdated or poorly maintained systems can introduce significant clinical risks by reducing image quality and increasing the likelihood of missed cancers or delayed diagnoses [35].

Low-resolution detectors may fail to reveal subtle but important features such as microcalcifications or spiculated margins. Aging equipment can introduce artefacts—such as linear streaks or image noise—that obscure or mimic pathology. Inconsistent performance from a lack of calibration further undermines diagnostic reliability.

**Strategies to ensure equipment quality:**

- A reliable, well-maintained mammography unit is the foundation of effective, high-quality breast imaging.
- Follow strict maintenance schedules and comprehensive quality assurance protocols.
- Adhere to regulations such as the Mammography Quality Standards Act (MQSA), which mandate routine performance testing and documentation.
- Promptly report and address equipment failures or imaging artefacts.

## Conclusion

Misdiagnosis of breast cancer on screening mammography remains a significant clinical challenge with major consequences for both patient care and medico-legal exposure. These diagnostic errors often stem from a combination of reader-related factors, lesion and patient characteristics, and technical limitations. Importantly, delayed diagnosis resulting from missed cancers can lead to larger tumor size, increased nodal involvement, more extensive surgery, and the need for additional adjuvant therapy, further impacting patient outcomes. However, such errors are not inevitable. Through targeted educational efforts, bias mitigation strategies, standardized protocols, and the integration of advanced technologies, radiologists can significantly reduce diagnostic errors. Incorporating peer learning and system-level changes fosters a culture of continuous improvement and accountability. By learning from past mistakes and proactively addressing the common pitfalls highlighted in this review, radiologists can enhance diagnostic precision and contribute to more timely and accurate breast cancer detection—ultimately improving outcomes for patients.

## Abbreviations

AI: Artificial intelligence
BI-RADS: Breast Imaging Reporting And Data System
CAD: Computer-assisted diagnosis
CC: Cranio-caudal
DBT: Digital breast tomosynthesis
MLO: Mediolateral oblique
MRI: Magnetic resonance imaging
